# Classification of Goat Vocalization via Lightweight Machine Learning and High-Dimensional Acoustic Features

**DOI:** 10.3390/ani16091394

**Published:** 2026-05-02

**Authors:** Daniel Alexander Méndez, Salvador Calvet Sanz

**Affiliations:** Institute of Animal Science and Technology, Universitat Politècnica de València, Camí de Vera s/n, 46022 Valencia, Spain; salcalsa@upvnet.upv.es

**Keywords:** precision livestock farming, caprine vocalization, animal welfare, bioacoustics, machine learning, deep learning

## Abstract

Continuous monitoring of livestock vocalizations offers a non-invasive tool for welfare assessment, but deploying current deep learning models in resource-constrained farm environments remains challenging due to high computational demands. This study proposes a feature-based machine learning pipeline optimized for edge computing to classify caprine vocalizations. Using the VOCAPRA dataset, which comprises 4147 labeled caprine vocalizations categorized into eight distinct welfare states and contexts, a hybrid feature extraction framework was applied to derive 156 spectral, temporal, and bioacoustic descriptors. Dimensionality reduction and a comprehensive comparative screening of 18 algorithms identified the CatBoost Classifier and a Multilayer Perceptron (MLP) as the optimal models. The CatBoost ensemble achieved a robust accuracy of 85.2%, while the optimized MLP reached 87.2% overall accuracy. An edge deployment benchmark revealed that the MLP was the best candidate with for real-time application, featuring a memory footprint of just 0.639 MB and near-instantaneous inference speeds of under 0.005 milliseconds per sample. Furthermore, feature importance and SHAP analyses revealed that mel-frequency cepstral coefficients heavily drove model decisions, particularly for identifying extreme physical distress and maternal reunion. The proposed methodology achieves competitive classification performance while dramatically reducing pre-processing and computational loads compared to image-based deep learning approaches, demonstrating the viability of lightweight-model, energy-efficient, real-time bioacoustic monitoring for precision livestock farming.

## Simple Summary

Nowadays, monitoring the well-being of farm animals continuously and without disturbing them is a major goal. For goats, vocalizations carry important information about their emotions, such as distress from an injury or heat, and more social-related information such as a mother recognizing her kid. While computers can learn to identify these sounds, current methods often require high computer capacity and are difficult to operate directly in barns or fields due to economical and energy constraints. This study developed a lightweight algorithm designed specifically to run on small, low-power devices directly on farms. By analyzing specific acoustic patterns in the sound waves rather than converting them into complex data, the algorithm successfully learned to recognize eight different goat behavioral contexts and states with high accuracy. The system was especially reliable at detecting extreme distress and mother–kid reunions. This development provides farmers with a practical, and real application tool to automatically monitor goat welfare in real-time, helping to identify animal conditions.

## 1. Introduction

Advancing on-farm welfare monitoring involves the development and validation of non-invasive, animal-based indicators of emotion that can be deployed at scale via precision livestock farming (PLF) systems [1,2]. PLF increasingly leverages bioacoustics to monitor animal welfare continuously and non-invasively. Acoustic data provides valuable information regarding the current state of the animal that sometimes cannot be detected by other modalities, such as movement sensors or computer vision [3]. Ruminant vocalizations, in particular, encode affective and welfare-relevant information, providing a robust channel for continuous welfare assessment across multiple farm environments [2,4,5,6,7,8,9,10]. The theoretical basis for this lies in the physiological mechanisms of sound production: emotional states directly alter vocal tract tension and respiration rates, resulting in measurable changes in acoustic parameters such as fundamental frequency, call duration, and energy distribution [11,12]. Thus, linking farm animal vocalizations to specific acoustic traits provides an objective measure of animal welfare.

Across livestock bioacoustics, resent research has predominantly followed two methodological streams to classify these acoustic traits. The first employs traditional or hybrid machine learning algorithms paired with engineered 1D acoustic features (e.g., MFCCs). For instance, recent real-time systems have combined MFCCs with explicit noise-filtering to achieve robust in-barn cattle monitoring [13], while similar hybrid pipelines in sheep successfully recognize feeding behaviors despite complex production noise [6,14,15]. The second stream utilizes deep learning models operating directly on 2D spectrograms or raw audio. While computationally intensive, recent innovations have optimized these deep architectures for commercial settings: lightweight CNNs now reliably detect distress calls in densely populated poultry houses [16], and advanced CNN-RNN hybrids support continuous, open-world cough monitoring in commercial swine facilities [17]. Furthermore, the field is actively addressing cross-farm generalizability through advanced data augmentation [18] and overcoming audio-only limitations via multimodal audio-visual fusion systems that localize specific events to individual animals in crowded pens [19].

Beyond single-species applications, modern PLF is increasingly intersecting with multi-species and multi-taxa acoustic monitoring to assess broader agroecosystem dynamics. Deploying these systems in heterogeneous agricultural soundscapes presents unique challenges, as high temporal variability and anthropogenic noise can easily confound traditional acoustic indices [20,21]. To handle this complexity, state-of-the-art approaches now utilize CNN-derived embeddings and Gaussian Mixture Models to filter uninformative audio across diverse agricultural land uses [21]. At the deployment level, systems are increasingly capable of multi-taxa monitoring at the edge, such as tracking bat populations via edge-AI in viticulture [3,22] and detecting multiple avian pest species using highly compressed, knowledge-distilled models designed for low-power microcontrollers [23]. These advances highlight a critical transition in bioacoustics: moving from controlled laboratory validations to robust, cross-environment deployment.

Within caprine bioacoustics specifically, vocalizations encode biologically meaningful information regarding both affective state and individual identity. For instance, Briefer et al. [7] demonstrated that goats in high-arousal states (such as those experiencing frustration) produce calls with higher fundamental frequencies and greater energy distribution. Environmental contexts also alter vocal response patterns; completely isolated dwarf goats emit calls characterized by more pronounced pureness and diminished randomness compared to those with partial social contact [24]. Foundational syntheses indicate that positive-valence goat calls tend to exhibit lower fundamental frequencies and reduced frequency modulation, establishing the biological validity of vocal cues as welfare indicators [5]. To operationalize these biological links, recent efforts have focused on developing deployable systems and standardized datasets [5,9]. A notable development is the public VOCAPRA corpus, developed to monitor acoustic environments across commercial dairy goat farms [9,10]. This comprehensive dataset comprises 4147 caprine vocalizations categorized into eight distinct welfare states and contexts, establishing a standardized baseline for robust benchmarking. Utilizing this dataset, recent studies employing explainable CNNs operating on time–frequency representations achieved high classification accuracy, illustrating significant performance advances in goat-focused models [9].

Despite these advances, transitioning from laboratory-scale models to continuous on-farm deployment presents significant hardware challenges. Edge deployment in rural areas is strictly constrained by energy availability and the computational limitations of microcontrollers [1,2]. This highlights a critical methodological gap in current caprine bioacoustics: the state-of-the-art relies on converting 1D audio signals into 2D spectrogram images for processing via CNNs [9]. While accurate, the computational overhead, high memory footprint, and intensive pre-processing requirements of these image transformations create a major bottleneck for real-time edge deployment. Currently, there is a lack of benchmarked, lightweight alternative pipelines specifically optimized for goat vocalizations that bypass this computationally expensive 2D step. This study addresses this limitation by proposing and validating an optimized, 1D feature-based machine learning pipeline explicitly designed for edge computing.

## 2. Materials and Methods

### 2.1. Dataset Preparation

The experiments were conducted using the publicly available Goat Bleatings dataset [25], collected within the framework of the VOCAPRA project [26]. The project consisted in a collection of basic data through a one-year-long data campaign, using dedicated devices (cameras and microphones) on the partner farms. Four dairy goat farms were involved, with one located in Lecco province with 105 lactating Alpine goats. The second one is located in the Oltrepó Pavese hills (Pavia province) with 64 lactating Alpine goats. The third one has 95 lactating Saanen goats. And the last farm hosts 29 Alpine goats [10]. While comprehensive details regarding the physical recording hardware, microphone specifications, and spatial positioning are extensively documented by Ntalampiras et al. [10], this study focuses only on the critical aspects of the acoustic data acquisition and curation process necessary for our machine learning pipeline.

To efficiently manage data storage by omitting silent periods, an onboard Acoustic Activity Detection algorithm processed the incoming audio through a biquadratic high-pass filter with a 200 Hz cutoff frequency and a Root Mean Square envelope follower. Audio recording was triggered whenever the sound envelope surpassed a dynamically updated adaptive threshold, capturing a one-second pre-trigger buffer and persisting for a five-second refractory period after the signal fell below the active threshold [10].

Subsequently, a data cleaning phase was implemented to isolate target goat bleats from ubiquitous environmental noises such as machinery, milking equipment, and human activity. This pre-filtering was achieved by deploying a detection algorithm based on the YAMNet deep neural network architecture to accurately identify and isolate relevant target sounds. Furthermore, a clean subset of vocalizations was extracted by applying a hierarchical clustering algorithm utilizing MFCCs to automatically segment the audio and separate pure bleat frames from residual background noise [10].

The labeling process was meticulously carried out by animal scientists utilizing a custom web-based annotation tool that cross-referenced the acoustic data with digital farm registers and synchronized video footage obtained from cameras installed within the barns. This multidimensional approach enabled the accurate categorization of the acoustic events into discrete contextual and state classes [10].

Finally, to prepare the curated dataset for the subsequent machine learning classification framework, each vocalization sample was standardized to a uniform two-second duration utilizing zero-padding where necessary [9]. The published dataset used in this work contains 4147 labeled vocalizations of goats categorized into the following eight classes:

- Heat: Reflecting the physiological and behavioral changes associated with reproductive cycles and mating.
- Feed distribution: Capturing vocalizations elicited during feeding times, shedding light on social dynamics and resource allocation within goat herds.
- Parturition: Documenting vocalizations during the birthing process, providing crucial insights into maternal behaviors and birthing rituals among goats.
- Injury or death: Documenting vocal expressions in response to injuries or mortality events, offering insights into distress signals and herd reactions to adverse events.
- Social isolation: Unveiling vocal cues associated with separation from herd members, highlighting the significance of social bonds and group cohesion among goats.
- Mother–kid reunion: Capturing vocal interactions upon reunion between mother goats and their kids, illuminating the significance of maternal bonds and social reunification in goat herds.
- Mother–kid separation: Observing vocalizations arising from the separation of mother goats and their offspring, elucidating maternal instincts and bonding dynamics.
- Presence of unknown visitors: Recording responses to unfamiliar human or animal presence, unveiling vigilance and territorial behaviors in farm settings.

**Figure 1:**
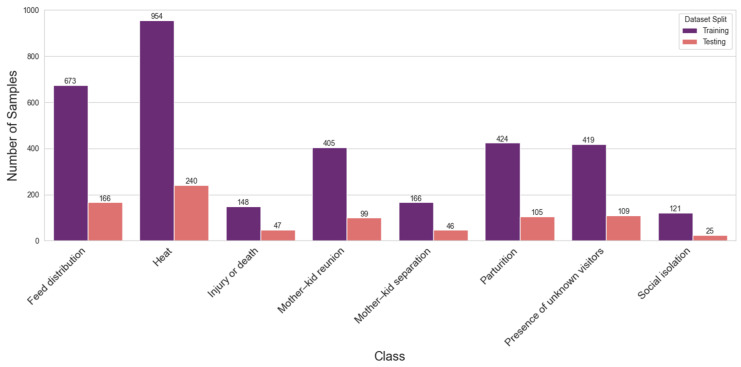
Data samples and distribution between train and test dataset.

As illustrated in Figure 1, the dataset exhibits significant class imbalance, reflecting the natural frequency of goat vocalizations in a farm setting. Categories such as heat (*N* = 1194) and feed distribution (*N* = 839) are predominant, whereas critical welfare indicators like social isolation (*N* = 146) and injury or death (*N* = 195) are comparatively rare. This method strictly maintained the relative class proportions within both subsets, ensuring that minority classes were adequately represented in the test set to prevent validity bias during model validation.

#### 2.1.1. Pre-Processing

The original audio recordings from the VOCAPRA dataset were captured at a sampling rate of 16 kHz. During the pre-processing phase, all audio files were upsampled to a uniform rate of 22,050 Hz. This standardization step was applied to align the data with the default operational parameters of the bioacoustic analysis libraries (e.g., Librosa) utilized in this study, ensuring mathematical stability and structural alignment for the subsequent calculation of Mel filter banks and spectral descriptors. Additionally, a secondary pre-processing protocol was implemented where the audio was upsampled to 30,000 Hz. This step was conducted as an empirical test to evaluate the effect of higher-resolution waveform interpolation on the stability of the feature extraction pipeline prior to model training.

#### 2.1.2. Feature Extraction

A hybrid feature extraction framework was developed to capture the multi-dimensional nature of the audio signals. Feature selection was grounded in the source–filter theory of mammalian vocal production and the specific acoustic challenges of precision livestock farming environments. To ensure reproducibility, all short-time Fourier transform (STFT) and subsequent temporal/spectral feature extractions were conducted using a standardized windowing process. A Hann window was applied with a frame size (window length) of 2048 samples and a hop length of 512 samples. In total, 156 descriptors were extracted, categorized by their biological and environmental relevance:

Vocal Tract Filtering and Timbre (MFCCs): Based on mammalian source–filter theory, MFCCs (20- and 40-coefficient filter banks) were extracted to model the resonant frequencies (formants) of the caprine vocal tract, which govern the timbral qualities necessary for individual identity and mother–kid recognition. The temporal dynamics of the signal, critical for distinguishing between stable and actively modulated calls, were encoded through the first-order (Delta (Δ)) and second-order (Delta-Delta (Δ Δ)) derivatives of the MFCCs [27].

Energy, Tension, and Arousal (Temporal and Pitch)**:** The fundamental frequency (F_0_) contour was extracted using the probabilistic pYIN algorithm [28]. In some mammals F_0_ is directly modulated by vocal fold tension, serving as a primary indicator of physiological arousal and stress. Autocorrelation was also computed to measure the tonal stability and pitch strength of the signal over time. To quantify the baseline physical effort and signal roughness, Root Mean Square (RMS) amplitude and Zero-Crossing Rate (ZCR) statistics were extracted. Furthermore, Spectral Flux and Temporal Centroid were calculated to distinguish impulsive attacks from sustained vocalizations [29].

Non-Linear Phenomena and Distress (Spectral Shape): High-arousal states frequently induce non-linear acoustic phenomena that transform tonal bleats into harsh, broadband screams. To capture these textural shifts, specific spectral descriptors were calculated. Spectral Centroid, Bandwidth, and Roll-off measure perceptual brightness, noise width, and the frequency skewness of the energy distribution, respectively. Spectral Contrast captures detailed peak–valley distinctions, while Spectral Entropy and Flatness are utilized to differentiate between highly organized tonal animal vocalizations and the chaotic broadband noise introduced by severe stress [28,29].

Vocalization Complexity and Bioacoustic Indices: Recognizing the specific requirements of bioacoustic analysis, high-level ecoacoustic indices were adapted to evaluate the structural properties of the calls. The Acoustic Complexity Index (ACI) was computed to quantify the intrinsic variability and amplitude modulation within the vocalizations themselves [30]. While traditionally used to filter environmental noise, within the context of isolated bleats, ACI serves as a direct measure of internal acoustic complexity; high-arousal or distress calls typically exhibit rapid, chaotic intensity fluctuations (resulting in a higher ACI), whereas calm contact or maternal calls maintain much steadier amplitude profiles. Additionally, the Bioacoustic Index was calculated by integrating the power spectrum strictly within the 2–8 kHz frequency band, capturing the energy distribution within the evolutionary range typically dominated by caprine vocal activity.

This comprehensive, biologically grounded feature vector ensures that the classifier has access to abstract spectral patterns, temporal evolution cues, and the biologically relevant anatomical characteristics of goat vocalizations (Table 1).

#### 2.1.3. Data Augmentation and Normalization

Following feature extraction, the dataset was partitioned into training (80%) and testing (20%) sets using stratified sampling. To address the inherent class imbalance (Figure 1), the Synthetic Minority Over-sampling Technique (SMOTE) was applied exclusively to the training partition, generating synthetic samples for minority classes to achieve a balanced distribution [31]. The test set remained unaugmented to ensure unbiased evaluation. Then, the feature vectors were standardized using Z-score normalization, scaling all descriptors to zero mean and unit variance to ensure numerical stability across different algorithms (Equation (1)).

(1) $$z=\frac{x-\mu }{\sigma }$$

where x is the original feature vector, μ is the mean of the training data, and σ is the standard deviation of the training data.

Given the high dimensionality of the extracted feature set (156 descriptors), a dimensionality reduction step was performed to identify the most discriminative attributes. This was achieved using sklearn’s SelectFromModel strategy with a LightGBM classifier as the underlying estimator. Instead of an iterative search, features were selected based on the importance weights assigned by the fitted LightGBM model, retaining only those that exceeded the selection threshold. This approach resulted in a compact, highly predictive feature subset that minimized computational complexity.

### 2.2. Model Development

#### 2.2.1. Classical Classifiers

An initial comparative screening was conducted using a comprehensive suite of algorithms, including tree-based ensembles (CatBoost, XGBoost, LightGBM, Random Forest, Extra Trees, AdaBoost, Gradient Boosting), linear models (Logistic Regression, Ridge, LDA), and others (SVM-Linear, QDA, Decision Tree, Naive Bayes, KNN). A Dummy Classifier was included to establish a baseline.

After selecting the best model, hyperparameter optimization was carried out using Optuna, an automatic hyperparameter optimization framework particularly designed for machine learning [32]. Unlike exhaustive grid search or simple random sampling, Optuna employs state-of-the-art algorithms, such as Tree-structured Parzen Estimator (TPE) for Bayesian optimization, to intelligently explore the search space by learning from previous trials and focusing on promising regions.

Each candidate configuration (a trial) was evaluated using k-fold cross-validation to ensure robustness and minimize overfitting. Additionally, Optuna supports pruning strategies that terminate unpromising trials early, significantly accelerating the search process without compromising solution quality [32].

#### 2.2.2. Multilayer Perceptron Implementation

To evaluate the predictive power of deep representations, a Multilayer Perceptron (MLP) feedforward neural network was developed. Unlike tree-based models, the MLP is sensitive to the scale of input data; therefore, prior to training, the 156-dimensional feature vector was standardized using Z-score normalization (zero mean and unit variance). To determine the optimal network architecture and hyperparameters, an exhaustive grid search with 3-fold cross-validation was implemented. The search space for the network topology evaluated various capacities, including single-layer (100 neurons), dual-layer (100–50, 254–58, and 256–128 neurons), and deeper three-layer (100–100–100 neurons) configurations. Both Rectified Linear Unit (ReLU) and hyperbolic tangent (tanh) activation functions were evaluated to introduce non-linearity, while the output layer utilized a Softmax function to produce probabilistic class predictions.

Model optimization was performed using the Adam solver, an algorithm for first-order gradient-based optimization of stochastic objective functions. The grid search systematically tuned the initial learning rate (0.0005, 0.001, and 0.01) alongside the batch size (32, 64, and 128 samples). To mitigate the risk of overfitting, a common challenge in high-dimensional bioacoustic data, two regularization strategies were strictly enforced. First, the L2 regularization penalty (alpha) was tuned across a range of values (0.0001, 0.001, 0.01, and 0.05) to penalize complex decision boundaries. Second, early stopping was configured to automatically terminate training if the validation loss failed to improve for 10 consecutive epochs. The models were permitted to train for a maximum of 500 iterations.

#### 2.2.3. Evaluation of Model Performance 

Performance indicators were estimated and derived from the confusion matrix, providing quantitative measures of model accuracy and reliability (Table 2). Since this study addresses a multiclass classification problem, these metrics were computed using the weighted-average approach. In this method, Precision, Recall, and the F-score were first computed for each class individually and then averaged across all classes, with each class’s contribution weighted by its frequency in the dataset. This approach ensured that the final evaluation accurately reflects the true distribution of the data, properly accounting for class imbalance without disproportionately amplifying the impact of minority classes.

Accuracy (Ac), Precision (P), Recall (R), Cohen’s Kappa (K), and the F-score range from 0 to 1, where higher values indicate better model performance. Accuracy represents the proportion of correctly classified instances across all categories, though it may be misleading in imbalanced datasets. Precision and Recall measure the ability to correctly identify positive instances, with higher values indicating fewer false positives and false negatives, respectively. Cohen’s Kappa accounts for agreement beyond chance, while the F-score, defined as the harmonic mean of Precision and Recall, balances both metrics and is particularly useful in class-imbalanced scenarios.

**Figure 2:**
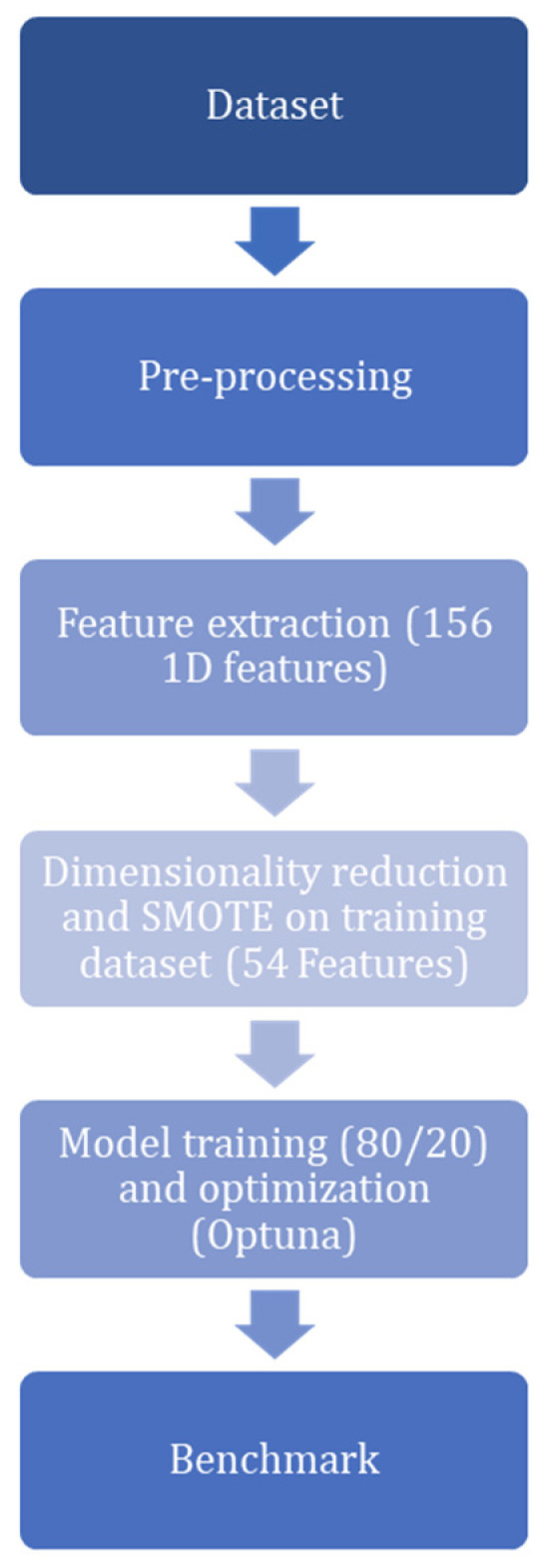
Diagram summarizing the data processing and machine learning pipeline implemented in this study.

Model generation, validation, and performance metrics were implemented in Python software version 3.11.7, using the PyCaret package version 3.4 [33] for classical machine learning models and the scikit-learn package version 1.8 for MLP development. All the operations were performed on a Windows-11 workstation. The workstation had a 32 GB intel i7-13700F 2.10 GHz Central Processing Unit (CPU), 32 GB of RAM and a 16 GB NVIDIA GeForce RTX 4060 Ti GPU. Figure 2 outlines the sequential phases of data processing, feature extraction, and model optimization implemented in this work.

## 3. Results

### 3.1. Data Characterization

**Figure 3:**
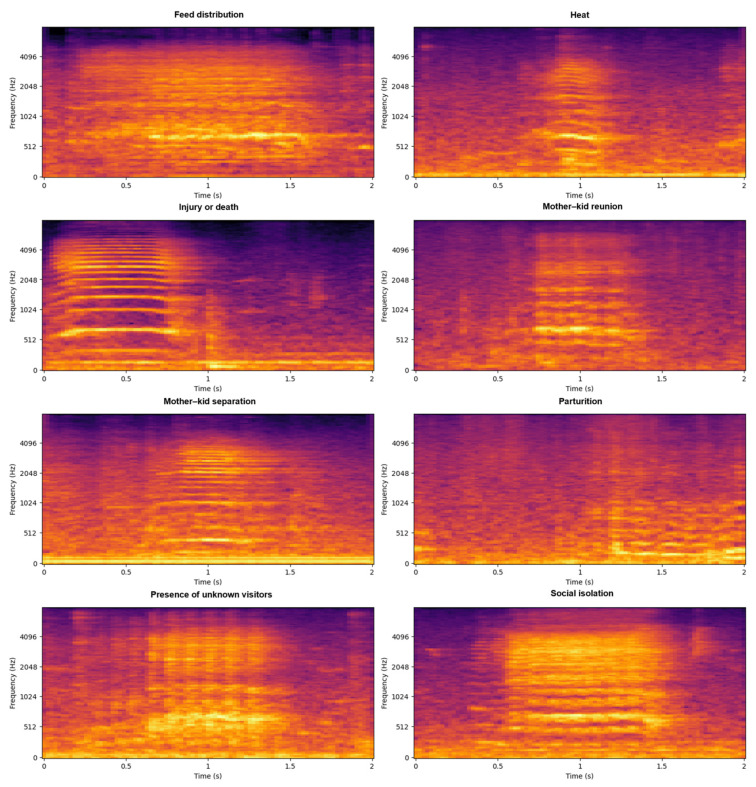
Spectrogram comparison across vocalization contexts. Each image depicts a single, representative acoustic sample selected to visually illustrate the hallmark structural differences characteristic of each respective class.

To visually illustrate the hallmark acoustic differences between behavioral categories, Figure 3 presents a single, representative spectrogram for each class. While individual spectrograms cannot capture the full intra-class acoustic variability inherent in biological signals, these specific samples were selected to demonstrate the fundamental structural extremes, ranging from highly tonal harmonics to chaotic broadband noise, that the subsequent high-dimensional feature extraction pipeline is designed to quantify. Notably, the representative acoustic profiles for injury or death and social isolation are characterized by distinct, widely spaced horizontal bands. This ladder-like harmonic structure reflects a strong fundamental frequency (F_0_) accompanied by prominent overtones, indicative of the sustained, highly tonal bleats typical of acute distress. Mathematically, the stark clarity of these harmonics generates a sharply defined spectral envelope, which consequently yields extreme magnitude values in the lower-order MFCCs. Similarly, maternal contact calls, such as mother–kid separation and mother–kid reunion, display clear harmonic structures consistent with sustained tonal bleating. However, these maternal spectrograms often exhibit greater frequency modulation, visible as wavy or contoured horizontal bands.

In contrast, the spectrograms for feed distribution and parturition lack clear horizontal stripes; instead, they appear as a “purple haze” or feature vertical smears, representing broadband noise. Behaviors triggered by acute external stimuli or herd excitement, such as the presence of unknown visitors, closely mirror this chaotic visual profile. These spectrograms exhibit jagged, broadband spectra with energy scattered across many frequency bins. Finally, vocalizations during heat present a hybrid acoustic visual; they contain fragmented harmonic partials heavily overlaid with broadband noise.

**Figure 4:**
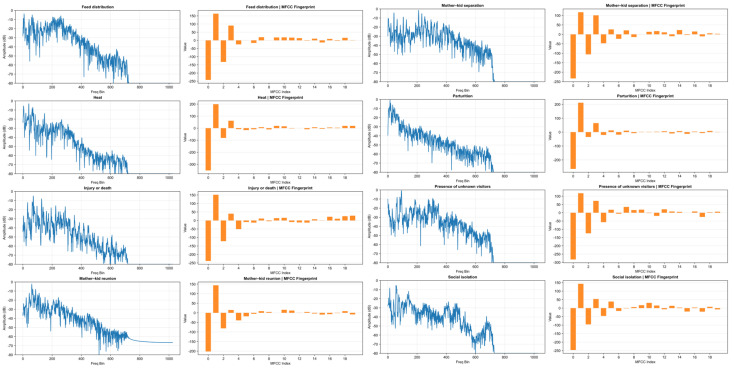
Peak energy frame analysis by of one sample by classes.

To identify distinct patterns for each class, MFCCs served as a robust, compact representation of the animal sound spectrum The bar charts visualize the exact input vectors provided to the models. The visual distinctness of these vectors, particularly the varying polarity and magnitude of indices 2, 4, and 5, confirms that the model has sufficient mathematical variance to distinguish between behaviors, even when the human ear or raw spectrogram might find them ambiguous (Figure 4).

The global MFCC profiles for each class demonstrate that while the overall spectral envelope follows a similar topological pattern across all behaviors, distinct amplitude variations are noticeable. Specifically, the first five coefficients (indices 0–4) (Figure 4) exhibit clear separation between classes, whereas higher-order coefficients tend to converge, indicating that the primary discriminative information is concentrated in the initial spectral components.

Other features also have a high participation in the differentiation of the vocalization classes that helps to improve the model capacity in the discrimination of the vocalization type. While global averages provide a trend, they risk smoothing out transient acoustic features. Figure 4 isolates the single frame of maximum energy for representative samples at 22,050 Hz, comparing the raw frequency spectrum (left column) with the extracted MFCC feature vector (right column). Behaviors such as feed distribution and presence of unknown visitors exhibit jagged, broadband spectra with energy distributed across many frequency bins. In contrast, social isolation and mother–kid separation display clearer harmonic structures (peaks at specific frequencies), consistent with the sustained, tonal bleating typical of contact calls. Even behaviors that appear similar in the spectrum produce distinct MFCC “fingerprints.” For example, comparing mother–kid separation to parturition, while both have high energy, separation exhibits a profound negative dip at MFCC index 2, whereas parturition shows a much shallower dip.

### 3.2. Model Development

In model development, the reduction in features for model prediction is a key factor in reducing the computing times for pre-processing previously to feeding the model for vocalization prediction. Feature selection via the LightGBM estimator successfully identified the most critical characteristics for accurate classification. This dimensionality reduction step not only refined predictive power but also optimized data processing and inference speeds for eventual edge deployment, ultimately retaining the 54 most important features for model evaluation.

With the selected features an initial screening of 18 machine learning algorithms was conducted to identify the optimal classifier. Prior to model selection, the effect of up sampling the audio to 3000 Hz was evaluated to test waveform interpolation stability; however, as it introduced no new biological frequency data beyond the original recording’s limits, it did not yield a noticeable performance gain. Conversely, utilizing a 40-coefficient MFCC filter bank (as opposed to a 20-coefficient one) provided an overall accuracy improvement of 2.19% (Tables S1–S4, Supplementary Materials), confirming that higher-order spectral information contains additional valuable information for vocalization differentiation.

Table 3 presents the performance ranking of the evaluated models. Tree-based ensembles demonstrated the strongest predictive capacity, achieving test set accuracies between 81% and 85%. While the CatBoost Classifier achieved the highest baseline accuracy (85.19%), the Light Gradient Boosting Machine (LightGBM) yielded near-identical performance (84.23%) with a small difference in computational architecture. Therefore, CatBoost was retained for subsequent analysis.

Hyperparameter tuning of the CatBoost model via the Optuna framework did not yield a noticeable performance improvement, achieving a marginal accuracy increase to 0.8542.

**Figure 5:**
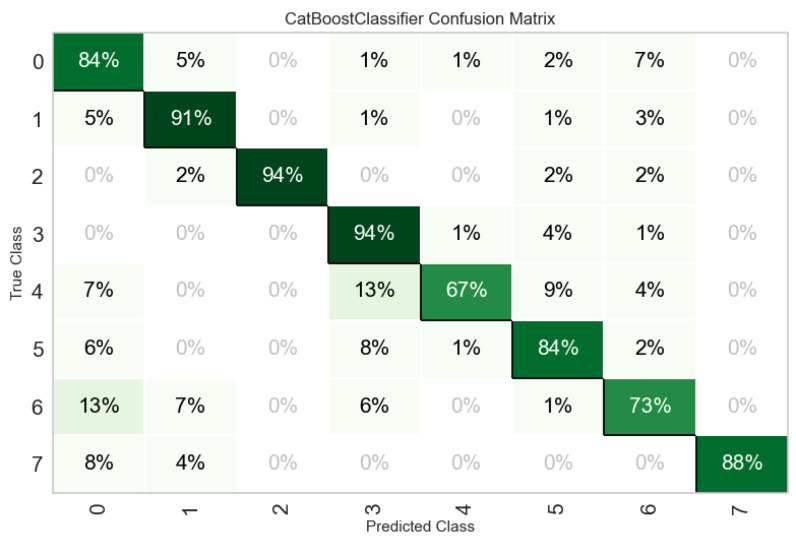
Normalized confusion matrix in the CatBoost model where feed distribution: 0; heat: 1; injury or death: 2; mother–kid reunion: 3; mother–kid separation: 4; parturition: 5; presence of unknown visitors: 6; social isolation: 7.

As the top-performing tree-based ensemble, the CatBoost Classifier demonstrated robust overall performance in categorizing goat vocalizations. However, an analysis of its confusion matrix (Figure 5) reveals that predictive accuracy varied significantly depending on the underlying biological and emotional state of the animals. The model exhibited exceptional accuracy in identifying distinct physiological or extreme emotional states. Calls related to injury or death and mother–kid reunion were the most accurately classified, both achieving a 94% true positive rate. Similarly, heat (91%) and social isolation (88%) were highly recognizable. Behaviors such as feed distribution (84%) and parturition (84%) were classified with high accuracy, though they exhibited slight acoustic overlap with other classes. For instance, parturition calls were occasionally misclassified as mother–kid reunion (8%) and feed distribution (6%).

The most notable misclassifications occurred in classes where emotional valence and arousal levels were structurally similar. The model struggled most with mother–kid separation (67%), which was frequently confused with other maternal and contact calls, specifically mother–kid reunion (13%) and parturition (9%). Likewise, vocalizations triggered by the presence of unknown visitors (73%) were frequently misclassified as feed distribution (13%) and heat (7%).

**Figure 6:**
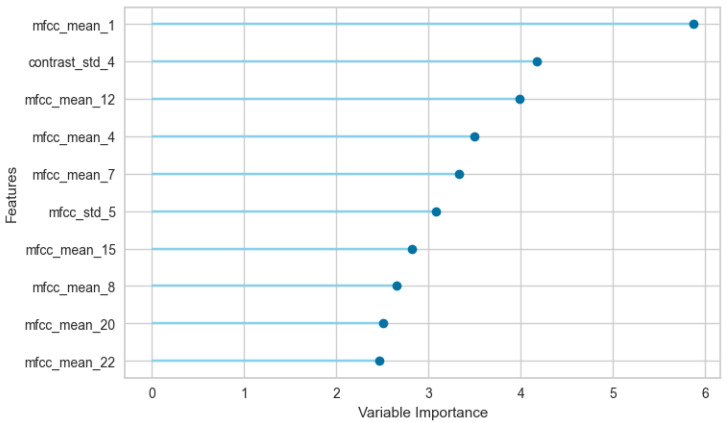
Feature importance rank on the CatBoost model classification.

A feature importance analysis was conducted on the CatBoost model to identify the most discriminative acoustic properties for caprine vocalization classification (Figure 6). The resulting variable importance plot shows a heavy reliance on MFCCs, though crucial standard deviation and spectral shape metrics also emerged at the top.

The first coefficient, mfcc_mean_1, emerged as the single most critical feature by a significant margin. However, the second most influential descriptor was not an MFCC, but rather contrast_std_4 (the standard deviation of spectral contrast in the fourth frequency band), highlighting the importance of textural peak–valley fluctuations over time.

The remainder of the top ten is dominated by a mix of lower- and middle-order MFCCs, specifically mfcc_mean_12, mfcc_mean_4, mfcc_mean_7, and mfcc_mean_8. The model also relies on the dynamic instability of the acoustic envelope, evidenced by the high ranking of mfcc_std_5. Furthermore, several higher-order coefficients ranked within the top tier—specifically mfcc_mean_15, mfcc_mean_20, and mfcc_mean_22—confirming that the network utilizes fine-grained micro-textures for state discrimination. Notably absent from the top predictors are standard temporal descriptors, fundamental frequency (pitch) statistics, and high-level ecoacoustic indices.

**Figure 7:**
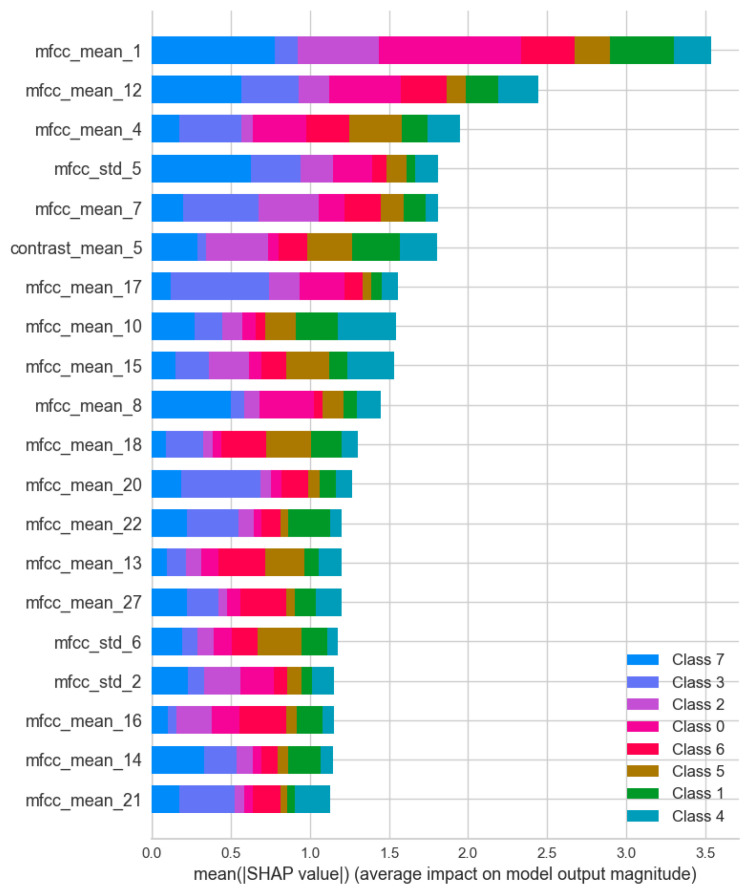
Feature importance by behavioral class derived from SHAP value analysis. Bar lengths denote the mean absolute SHAP value of the top acoustic predictors, where classes are: feed distribution: 0; heat: 1; injury or death: 2; mother–kid reunion: 3; mother–kid separation: 4; parturition: 5; presence of unknown visitors: 6; social isolation: 7.

To achieve a granular understanding of feature contributions across individual classes, a SHAP value analysis was performed on the selected CatBoost model (Figure 7.). While the global feature importance plot identifies overarching predictors like mfcc_mean_1 and contrast_std_4, the SHAP summary (Figure 7) reveals highly specialized, behavior-specific acoustic drivers.

mfcc_mean_1 maintains its position as the most impactful feature globally in the SHAP analysis, showing significant predictive contributions across almost all behaviors, particularly for feed distribution and social isolation.

Interestingly, while the standard deviation of contrast dominated the global metric, the SHAP analysis highlights contrast_mean_5 as the primary textural driver for individual predictions. The SHAP plot demonstrates that the impact of this feature is dedicated to predicting a single category: injury or death. Similarly, mfcc_mean_7 exhibits a disproportionately high impact on injury or death compared to other behaviors,

Other classes also exhibit distinct acoustic dependencies. Social isolation is mainly driven by the variance in the fifth cepstral coefficient (mfcc_std_5), alongside mfcc_mean_12 and mfcc_mean_8. Mother–kid reunion is heavily influenced by higher-order coefficients, notably mfcc_mean_17, mfcc_mean_20, and mfcc_mean_21. Meanwhile, feed distribution and heat relied on a broader mix of primary MFCCs (e.g., mfcc_mean_4).

### 3.3. Artificial Neural Network Implementation

The grid search identified the optimal MLP architecture as a deep, two-hidden-layer configuration containing 256 and 128 neurons, respectively. The hyperbolic tangent (tanh) activation function yielded higher performance compared to the standard ReLU, operating alongside the Adam solver initialized with a learning rate of 0.001. A smaller batch size of 32 samples and an L2 regularization penalty (alpha) of 0.001 were selected to balance gradient update frequency and model generalization.

**Figure 8:**
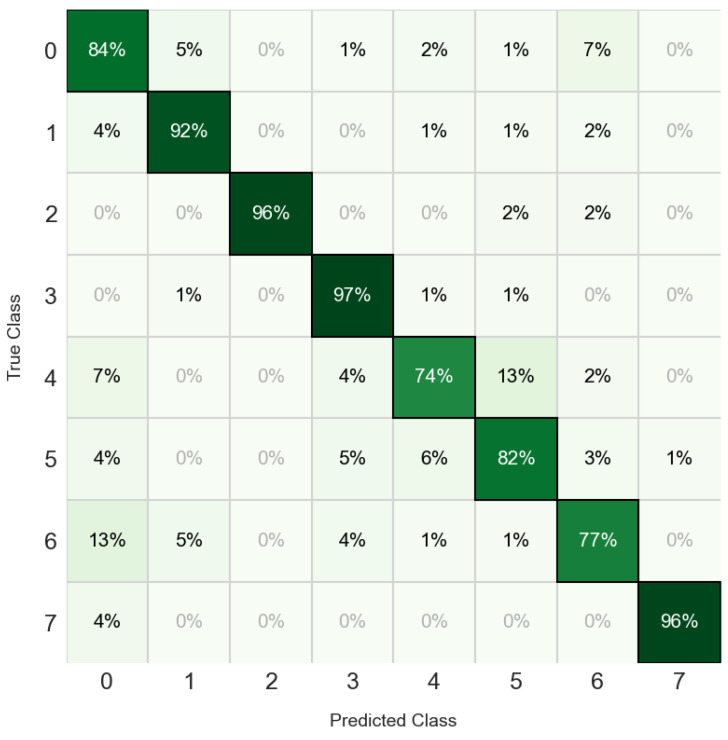
Normalized confusion matrix in the Multilayer Perceptron model where classes are: feed distribution: 0; heat: 1; injury or death: 2; mother–kid reunion: 3; mother–kid separation: 4; parturition: 5; presence of unknown visitors: 6; social isolation: 7.

Evaluated on the testing partition, the optimized MLP achieved a high overall classification accuracy of 87.2% (Figure 8). An analysis of the confusion matrix reveals improved predictive precision across critical distress and social categories. The model correctly classified 97.0% of mother–kid reunion calls, 96% of injury or death vocalizations, and 92% (224 out of 240) of heat events. Social isolation also demonstrated strong discriminability, achieving 92.0% (23 out of 25) accuracy.

Moderate performance was observed for routine biological events, with feed distribution and parturition reaching accuracies of 84% and 82%, respectively. The lowest classification accuracies were recorded for presence of unknown visitors (77%) and mother–kid separation (74%). Notably, 14 instances of unknown visitor calls were misclassified as feed distribution, while mother–kid separation calls were occasionally confused with parturition (5 instances) and reunion (4 instances).

**Figure 9:**
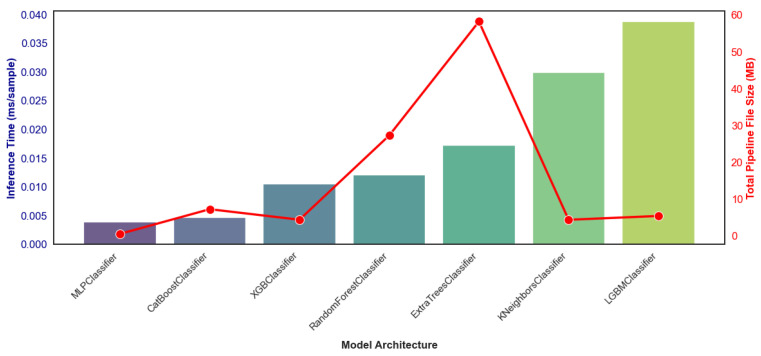
Benchmark comparing true inference latency (left axis, bars) and total memory footprint (right axis, line) across candidate model architectures.

To determine the most suitable architecture for resource-constrained hardware, a baseline algorithmic efficiency benchmark was conducted evaluating inference latency and total memory footprint (Figure 9). To isolate the computational efficiency of the models themselves, latency measurements were obtained using a standardized hardware environment (Intel Core i7-13700F CPU @ 2.10 GHz, 32 GB RAM). This benchmark revealed a stark contrast in operational efficiency among the models prior to physical micro-controller deployment. Tree-based ensembles such as Random Forest and Extra Trees showed the higher memory footprints, reaching approximately 30 MB and 60 MB, respectively. Conversely, while the LightGBM model maintained a compact file size (~6 MB), it exhibited the highest inference latency in the test group, averaging roughly 0.038 ms per sample.

Ultimately, the MLP emerged as the architecture for potential edge deployment. The MLP model drastically outperformed all ensemble methods, achieving near-instantaneous inference speeds of under 0.005 ms per sample. Furthermore, the complete MLP pipeline required a minimal file size of just 0.639 MB. This represents a dramatic reduction in both computational time and memory requirements compared to the previous models.

## 4. Discussion

Previous studies in caprine bioacoustics have principally implemented deep learning approaches focused on Convolutional Neural Networks (CNNs). These pipelines require intensive pre-processing, including the transformation of 1D audio signals into 2D spectrogram images, extensive data segmentation for image homogenization, and complex augmentation strategies to balance classes [9,34]. While these lab-scale approaches are promising, reaching average classification accuracies ranging from 68.3% (MobileNet) to 95.8% (custom models) and 82.75% for VGGish-based architectures [9,34], they introduce significant computational overload.

In contrast, the presented approach implements a straightforward, lightweight pipeline with reduced pre-processing steps. Unlike approaches that rely on converting audio into image-like data for CNNs, this method avoids computationally intensive transformations, drastically lowering the computational burden associated with pre-processing and model inference. By relying on a high-dimensional 1D feature vector rather than a 2D one, our optimized MLP (87.2%) and CatBoost (85.2%) models achieve performance that is highly competitive with these deep architectures [9]. Furthermore, this performance aligns closely with high-accuracy traditional machine learning baselines (e.g., Random Forest, k-NN) recently benchmarked in cattle and sheep studies [35,36]. This methodology achieves highly average classification accuracies while dramatically reducing the computational load. This trade-off is particularly advantageous for data inference and continuous edge deployment in farm environments. The hyperparameter optimization of the MLP further supports this; the selection of the tanh activation function and a deep architecture (256–128 neurons) confirms that while caprine acoustic features require complex, non-linear hierarchical transformations for effective separation, this can be achieved without defaulting to resource-heavy image type classification networks.

### 4.1. Acoustic Drivers and Biological Signatures of Caprine Behavior

The feature importance and SHAP value analyses bridge the gap between machine learning mechanics and caprine bioacoustics, revealing that the classifier relies heavily on the complex timbral qualities and spectral envelopes of the bleats. The high importance of mfcc_mean_1 aligns with computational bioacoustic principles, as it correlates with the overall spectral energy balance and broad resonance of the vocal tract [37]. Furthermore, research in livestock bioacoustics indicates that emotional states directly alter vocal tract tension and respiration rates, which mathematically manifests in these lower-order cepstral coefficients [1,11,12,38].

However, different emotional and physiological states are governed by entirely different, specific acoustic phenomena. For extreme distress, the model almost exclusively relies on contrast_mean_5 and contrast_std_4 to identify injury or death calls, identifying a close biological relation. While standard goat contact bleats are highly tonal with clear harmonic peaks, severe pain vocalizations often feature non-linear acoustic phenomena, such as deterministic chaos, that flood the sound wave with broadband noise [39,40]. The algorithm successfully learned to associate this drastic alteration in spectral contrast with extreme physical distress, confirming that the model isolates extreme distress using specific mid-frequency structural and contrast anomalies.

In the case of individual recognition and reunion, vocalization such as mother–kid reunion was mainly driven by higher-order MFCCs (e.g., mfcc_mean_20, mfcc_mean_21 and mfcc_mean_17). Because reunion relies heavily on mutual individual recognition, these calls must carry a distinct “vocal signature” [37,41]. The model’s reliance on these higher-order coefficients, which encode subtle acoustic micro-textures, suggests it utilizes precise, individualistic timbral cues akin to recognizing a specific human voice.

Conversely vocalizations like social isolation were heavily influenced by mfcc_std_5. Bioacoustically, isolation induces psychological stress, which has been explicitly shown to alter the pureness and increase the randomness of dwarf goat calls [24]. The high predictive power of this standard deviation indicates that dynamic instability in the call’s timbre is a key bioacoustic marker of separation anxiety. Conversely, classes like feed distribution and states heat relied on a broader mix of primary lower-order MFCCs. Because these lower-order coefficients mathematically encode the broad spectral envelope (formants), this indicates that these calls are differentiated by macroscopic structural shifts in the vocal tract, such as changes in articulation or tract length during estrus, rather than isolated textural anomalies [42,43].

Likewise, the emergence of higher-order coefficients (e.g., mfcc_mean_17, 20, 21, and 22) empirically validates the methodological decision to extract a high-dimensional MFCC vector (40 coefficients) rather than the standard 13 or 20 coefficients. Distinct welfare states are communicated through complex, high-frequency timbral shifts, confirming that higher-resolution feature extraction is necessary for precision monitoring.

### 4.2. Biological Constraints and Acoustic Overlap

While the MLP’s classification performance aligns tightly with the evolutionary distinctiveness of critical states (injury, reunion), the model’s specific areas of confusion reflect the acoustic overlap inherent in shared states of physiological arousal. One of the most difficult behavioral boundaries to classify was found among heat, feed distribution, and the presence of unknown visitors. Calls triggered by human presence were frequently misclassified as feeding anticipation or heat. Acoustically, these calls share the increased acoustic energy, higher fundamental frequencies, and broad bandwidths typically seen in high-arousal states [7,12]. This reflects the combination of extreme vocal effort and intense physical arousal characteristic of both estrus and acute alarm [11]. Furthermore, this broadband spectral profile correlates with the “chaotic” nature of chewing or herd movement noises, successfully capturing the chaotic nature of high-arousal alarm calls that confuse the algorithm’s decision boundaries.

Furthermore, persistent overlap within maternal contexts (e.g., separation misclassified as parturition or reunion) highlights a fundamental challenge in bioacoustics. Separation, parturition, and reunion all heavily involve epimeletic and etepimeletic (care-giving and care-seeking) vocalizations [44,45,46,47]. These calls share fundamental frequency contours and timbral qualities designed for mother–offspring communication and individual recognition. Because the internal biological mechanism of a mother vocalizing for her offspring remains acoustically conserved even as the external context shifts, purely acoustic classifiers face inherent biological limits when separating these states.

### 4.3. Translation to Functional Welfare Assessment

While the MLP achieved an 87.2% overall accuracy across the eight distinct behavioral classes, evaluating the model strictly on class-level performance underrepresents its true utility for animal welfare monitoring. From a practical precision livestock farming (PLF) perspective, identifying the exact behavioral label is often less critical than detecting shifts in the animals’ underlying emotional valence (positive vs. negative states) and physiological arousal (low vs. high energy) [2,38].

As noted in the analysis, the model frequently confused calls related to heat, feed distribution, and the presence of unknown visitors. From a functional welfare perspective, all three classes reflect states of high physiological arousal and heightened environmental interaction. If an automated PLF system triggers a generic “high arousal” or “herd disturbance” alert based on these overlapping acoustics, the stockperson is successfully informed of a significant herd-level event requiring attention [2,48].

Conversely, the model demonstrated its highest precision in isolating critical negative-valence, high-arousal states that require immediate intervention. Vocalizations indicating injury or death (96% accuracy) and social isolation (92% accuracy) were cleanly separated from routine herd noise and other arousal states. The ability of acoustic descriptors to accurately isolate high-arousal distress from general vocalizations aligns with established bioacoustic mechanisms [11]. In the context of welfare monitoring, these represent the most urgent “red flag” events. The model’s ability to prioritize and accurately identify acute physical or psychological distress without confusing them with general arousal confirms that its practical utility as a stress-detection tool exceeds its nominal 87.2% mathematical accuracy.

Therefore, deploying this lightweight pipeline in a real-world farm environment does not necessarily require perfect multi-class discrimination. By aggregating these eight behavioral classes into functional welfare categories, such as routine communication, general arousal, and acute distress, the proposed framework provides a highly reliable, computationally efficient mechanism for continuous welfare quantification, confirming that the MLP provides the optimal balance of high predictive accuracy and extreme efficiency required for real-time classification on microcontroller-based PLF systems.

### 4.4. Limitations and Future Work

While the current study demonstrates the efficacy of lightweight machine learning models and high-dimensional MFCCs for the classification of caprine vocalizations, several limitations must be acknowledged. First, the inherent acoustic overlap between shared states of physiological arousal, such as the confusion between feeding anticipation and the presence of unknown visitors, or overlapping maternal care vocalizations, indicates a biological upper limit to unimodal audio classification. When the internal physiological mechanism of a vocalization is conserved despite differing external contexts, purely acoustic classifiers will inevitably face decision boundary confusion. Second, the current classification pipeline was evaluated offline using discrete, pre-processed audio segments. This static approach does not fully capture the complex, overlapping noise dynamics and continuous acoustic streams of a real-world farm environment.

To address these limitations, future research will focus on transitioning this framework into a robust, real-time PLF deployment. This transition will require specific architectural advancements to bridge the gap between static file analysis and live monitoring.

To handle uninterrupted audio streams effectively, future implementations will utilize a sliding window ring buffer coupled with temporal smoothing. Aggregating predictions over a short temporal horizon using majority voting will be essential to mitigate the “flickering” effect common in frame-by-frame classification and to reduce transient false positives caused by unpredictable farm noise.

To overcome the biological constraints of acoustic overlap, future systems must integrate the audio inference engine with concurrent visual data streams. By employing a labeling template for video object tracking, the framework will achieve multimodal contextual awareness. In this proposed hybrid architecture, the audio pipeline can be dynamically gated or contextualized by visual detection; for instance, specific audio classifications can be verified against goat movements, postures, or proximities identified by the visual tracker. This fusion will optimize computational resources on edge devices while providing the broader behavioral context necessary to definitively resolve acoustically ambiguous vocalizations.

Third, the proposed pipeline relies on the global extraction of 1D acoustic features from isolated audio segments. In high-density farm environments where multiple animals vocalize simultaneously, overlapping calls will inherently distort the global spectral envelope and the calculated MFCCs, likely leading to misclassification or reduced confidence scores. Therefore, future iterations of this framework must incorporate robust blind source separation (BSS) algorithms or spatial acoustic filtering (e.g., microphone arrays) prior to feature extraction to isolate individual vocalizations from concurrent herd noise.

Additionally, future work should focus on the challenges that may arise from differences in breeds and production systems. Evaluating models across diverse farms would strengthen their generalization capacity and help determine whether fine-tuning is required for specific deployment scenarios.

## 5. Conclusions

This study provides evidence that a lightweight, feature-based machine learning pipeline can effectively classify caprine vocalizations across diverse classes, offering a computationally efficient alternative to image-based deep learning architectures. By extracting a comprehensive 1D acoustic feature vector, the optimized CatBoost ensemble and Multilayer Perceptron (MLP) models achieved robust classification accuracies of 85.2% and 87.2%, respectively. Feature importance and SHAP analyses empirically validated this approach, revealing that both lower and higher-order MFCCs, alongside specific spectral contrast variations, act as the primary acoustic drivers for state discrimination. The models demonstrated high sensitivity to distinct biological and emotional states, effectively isolating the chaotic spectral signatures of severe physical distress (injury) and the highly individualized, complex timbral cues associated with mother–kid reunions.

The presented framework validates the feasibility of continuous, non-invasive welfare monitoring on resource-constrained hardware. The benchmark revealed that the MLP provides the optimal architectural balance. It demonstrated notable efficiency advantages over the evaluated methods, combining high predictive precision with a minimal memory footprint (<1 MB) and near-instantaneous, sub-millisecond inference latency. However, these findings must be interpreted within the context of the study’s limitations. While the models achieved high accuracy on the standardized VOCAPRA corpus, their generalization capacity across different goat breeds, varying farm acoustic environments, and diverse production systems remains to be validated. Furthermore, the inherent acoustic overlap between vocalizations triggered by shared states of physiological arousal or conserved maternal mechanisms suggests a biological upper limit to purely unimodal audio classification. Despite these constraints, this study establishes a strong methodological foundation for on-farm deployment. Future integration of this lightweight acoustic pipeline with temporal smoothing and multimodal sensor fusion will be essential to bridge the gap between static sound classification and real-time, context-aware precision livestock farming.

## Figures and Tables

**Table 1:** Summary of the 156 acoustic features extracted for model development. The “Count” column indicates the total number of individual statistical measures (e.g., means, standard deviations, or discrete coefficients) generated for each specific feature category.

Domain	Feature Name	Function/Description	Count
Temporal & Energy	RMS Statistics	Mean and standard deviation (Std) of Root Mean Square amplitude. Measures loudness and dynamic range.	2
	ZCR Statistics	Mean and Std of Zero-Crossing Rate. Indicators of signal “roughness” or noisiness.	2
	Autocorrelation	Peak of the autocorrelation function. Indicates pitch strength/periodicity (tonal stability).	1
Time Evolution	Spectral Flux	Mean and Std of Onset Strength. Measures the rate of change in the spectrum (stability vs. chaos).	2
	Temporal Centroid	Center of gravity of the signal in time. Distinguishes impulsive sounds (attacks) from sustained calls.	1
Spectral Timbre	MFCC Statistics	Mean and Std of the first 40 coefficients. Captures the detailed static spectral envelope (identity).	40
Dynamics	Δ MFCC Stats	Mean and Std of the 1st derivative (Velocity) for all 40 coefficients. Captures the rate of timbral change.	40
	ΔΔ MFCC Stats	Mean and Std of the 2nd derivative (Acceleration) for all 40 coefficients. Captures the complexity of transitions.	40
Spectral Shape	Centroid	Mean and Std. Center of mass of the spectrum. Correlates with perceptual “brightness”.	2
	Bandwidth	Mean and Std. Spectral width. Distinguishes tonal sounds from broadband noise.	2
	Roll-off	Mean and Std. Frequency below which 85% of energy is contained.	2
	Contrast	Mean and Std calculated across 7 frequency bands. Measures peak–valley distinction (texture).	14
Complexity	Entropy & Flatness	Spectral Entropy (disorder) and Spectral Flatness (noisiness). Distinguishes noise from tone.	2
Bioacoustic	ACI	Acoustic Complexity Index. Quantifies intensity variability to filter anthropogenic noise.	1
	Bioacoustic Index	Total energy in the 2–8 kHz band. Specific to bird/mammal vocalization range.	1
Pitch (pYIN)	F_0_ Statistics	Mean, Max, Std, and Slope of the Fundamental Frequency (F_0_). Captures intonation and stress.	4

**Table 2:** Metrics used to evaluate the model’s performance.

Metric	Description	
Accuracy (Ac)	$\frac{\left(TP+TN\right)}{\left(TP+TN+FP+FN\right)}$	(2)
Precision (P)	$\frac{TP}{TP+FP}$	(3)
Recall (R)	$\frac{TP}{TP+FN}$	(4)
Cohen’s Kappa (K)	$\frac{\mathrm{Pr}\left(a\right)-Pr(e)}{1-Pr(e)}$	(5)
F1-score	$\frac{2\;*\;P\;*\;R}{P+R}$	(6)

True positives (TP) represent correctly predicted classes, while false negatives (FN) occur when observed classes are misclassified. False positives (FP) arise when unobserved classes are predicted, and true negatives (TN) correspond to classes that were neither observed nor predicted. For Cohen’s Kappa calculation, the term Pr(a) refers to the observed agreement between predictions and actual values, while Pr(e) represents the expected agreement by chance.

**Table 3:** Ranking classification order according accuracy of the 18 models evaluated to predict the classes from feature extraction.

Model	Accuracy	Recall	Prec.	F1-Score	Kappa
CatBoost Classifier	0.8519	0.8519	0.8529	0.8508	0.8199
Light Gradient Boosting Machine	0.8423	0.8423	0.8431	0.8412	0.8077
Extreme Gradient Boosting	0.8363	0.8363	0.8364	0.8357	0.8009
Extra Trees Classifier	0.8268	0.8268	0.8306	0.8261	0.789
K Neighbors Classifier	0.822	0.822	0.8336	0.8241	0.7866
Random Forest Classifier	0.8184	0.8184	0.8218	0.818	0.7792
Quadratic Discriminant Analysis	0.8184	0.8184	0.8274	0.815	0.7772
Gradient Boosting Classifier	0.779	0.779	0.7792	0.7784	0.7322
Logistic Regression	0.724	0.724	0.7405	0.729	0.6694
Linear Discriminant Analysis	0.7085	0.7085	0.7316	0.7138	0.6521
Ridge Classifier	0.6965	0.6965	0.7209	0.7006	0.6389
SVM–Linear Kernel	0.6822	0.6822	0.7211	0.6922	0.6229
Naive Bayes	0.5902	0.5902	0.6204	0.5944	0.513
Decision Tree Classifier	0.5532	0.5532	0.5662	0.5576	0.4639
Ada Boost Classifier	0.2306	0.2306	0.3102	0.2209	0.134
Dummy Classifier	0.1983	0.1983	0.0393	0.0656	0

## Data Availability

Data used is freely available at https://zenodo.org/records/7530401 (accessed on 25 November 2025).

## Supplementary Materials

The following supporting information can be downloaded at https://www.mdpi.com/article/10.3390/ani16091394/s1. Table S1: Ranking classification order according accuracy of the 18 models evaluated to predict vocalization behavior form feature extraction using 20-coefficient MFCC and sampling of 22,050 Hz; Table S2: Ranking classification order according accuracy of the 18 models evaluated to predict vocalization behavior form feature extraction using 20-coefficient MFCC and sampling of 30,000 Hz; Table S3: Ranking classification order according accuracy of the 18 models evaluated to predict vocalization behavior form feature extraction using 40-coefficient MFCC and sampling of 22,050 Hz; Table S4. Ranking classification order according accuracy of the 18 models evaluated to predict vocalization behavior form feature extraction using 40-coefficient MFCC and sampling of 30,000 Hz.
