# Supplementary material for: Classification of Goat Vocalization via Lightweight Machine Learning and High-Dimensional Acoustic Features
Source: Animals (Basel). 2026 May 2;16(9):1394. doi: 10.3390/ani16091394 (PMC13163017; doi:10.3390/ani16091394)
Supplement: Supplementary file 1 [file animals-16-01394-s001.zip › animals-4260209-supplementary.pdf]

Supplementary material

Classification of Goat Vocalization via Lightweight Machine Learning and High-Dimensional Acoustic Features

Daniel Alexander Méndez Reyes [1,*], Salvador Calvet Sanz[1]

[1] Institute of Animal Science and Technology, Universitat Politècnica de València, Camí de Vera, 46022 Valencia, Spain, damenre@upv.es
* Correspondence: damenre@upv.es;

Table S1. Ranking classification order according accuracy of the 18 models evaluated to predict vocalization behavior form feature extraction using 20-coefficient MFCC and sampling of 22050 Hz.

Model	Accuracy	Recall	Prec.	F1	Kappa
CatBoost Classifier	0.830	0.830	0.830	0.829	0.7939
Extra Trees Classifier	0.826	0.826	0.830	0.824	0.7873
K Neighbors Classifier	0.811	0.811	0.830	0.814	0.7744
Light Gradient Boosting Machine	0.811	0.811	0.812	0.811	0.7701
Extreme Gradient Boosting	0.809	0.809	0.811	0.809	0.7676
Random Forest Classifier	0.801	0.801	0.803	0.799	0.7573
Quadratic Discriminant Analysis	0.779	0.779	0.786	0.780	0.7318
Gradient Boosting Classifier	0.738	0.738	0.746	0.741	0.6848
Logistic Regression	0.649	0.649	0.678	0.655	0.5836
Linear Discriminant Analysis	0.633	0.633	0.665	0.638	0.5664
SVM - Linear Kernel	0.621	0.621	0.656	0.628	0.5523
Ridge Classifier	0.597	0.597	0.641	0.601	0.5269
Decision Tree Classifier	0.565	0.565	0.575	0.567	0.4775
Naive Bayes	0.548	0.548	0.586	0.555	0.4671
Ada Boost Classifier	0.339	0.339	0.418	0.333	0.2393
Dummy Classifier	0.1983	0.1983	0.0393	0.0656	0

Table S2. Ranking classification order according accuracy of the 18 models evaluated to predict vocalization behavior form feature extraction using 20-coefficient MFCC and sampling of 30000 Hz.

Model	Accuracy	Recall	Prec.	F1	Kappa
CatBoost Classifier	0.8483	0.8483	0.8481	0.8476	0.8157
Light Gradient Boosting Machine	0.8136	0.8136	0.8144	0.8128	0.7728
K Neighbors Classifier	0.8124	0.8124	0.8232	0.8144	0.7747
Extra Trees Classifier	0.8112	0.8112	0.8142	0.8097	0.7699
Extreme Gradient Boosting	0.8088	0.8088	0.8099	0.8086	0.7674
Random Forest Classifier	0.7993	0.7993	0.8	0.7976	0.7553
Quadratic Discriminant Analysis	0.7849	0.7849	0.7881	0.7853	0.7386
Gradient Boosting Classifier	0.7467	0.7467	0.7527	0.7486	0.6941

Logistic Regression	0.6464	0.6464	0.6734	0.6537	0.579
Linear Discriminant Analysis	0.6356	0.6356	0.6809	0.6463	0.57
SVM - Linear Kernel	0.6344	0.6344	0.6664	0.6396	0.5655
Ridge Classifier	0.6249	0.6249	0.6638	0.6294	0.558
Naive Bayes	0.5723	0.5723	0.6042	0.5782	0.4915
Decision Tree Classifier	0.5723	0.5723	0.5912	0.5782	0.4875
Ada Boost Classifier	0.4134	0.4134	0.4815	0.4241	0.3119
Dummy Classifier	0.1983	0.1983	0.0393	0.0656	0

Table S3. Ranking classification order according accuracy of the 18 models evaluated to predict vocalization behavior form feature extraction using 40-coefficient MFCC and sampling of 22050 Hz.

Model	Accuracy	Recall	Prec.	F1	Kappa
CatBoost Classifier	0.8519	0.8519	0.8529	0.8508	0.8199
Light Gradient Boosting Machine	0.8423	0.8423	0.8431	0.8412	0.8077
Extreme Gradient Boosting	0.8363	0.8363	0.8364	0.8357	0.8009
Extra Trees Classifier	0.8268	0.8268	0.8306	0.8261	0.789
K Neighbors Classifier	0.822	0.822	0.8336	0.8241	0.7866
Random Forest Classifier	0.8184	0.8184	0.8218	0.818	0.7792
Quadratic Discriminant Analysis	0.8184	0.8184	0.8274	0.815	0.7772
Gradient Boosting Classifier	0.779	0.779	0.7792	0.7784	0.7322
Logistic Regression	0.724	0.724	0.7405	0.729	0.6694
Linear Discriminant Analysis	0.7085	0.7085	0.7316	0.7138	0.6521
Ridge Classifier	0.6965	0.6965	0.7209	0.7006	0.6389
SVM - Linear Kernel	0.6822	0.6822	0.7211	0.6922	0.6229
Naive Bayes	0.5902	0.5902	0.6204	0.5944	0.513
Decision Tree Classifier	0.5532	0.5532	0.5662	0.5576	0.4639
Ada Boost Classifier	0.2306	0.2306	0.3102	0.2209	0.134
Dummy Classifier	0.1983	0.1983	0.0393	0.0656	0

Table S4. Ranking classification order according accuracy of the 18 models evaluated to predict vocalization behavior form feature extraction using 40-coefficient MFCC and sampling of 30000 Hz.

Model	Accuracy	Recall	Prec.	F1	Kappa
CatBoost Classifier	0.8554	0.8554	0.8552	0.8546	0.8246
Extreme Gradient Boosting	0.8447	0.8447	0.844	0.8438	0.811
Light Gradient Boosting Machine	0.8327	0.8327	0.833	0.8308	0.7957
K Neighbors Classifier	0.8303	0.8303	0.8442	0.8335	0.7967
Random Forest Classifier	0.8208	0.8208	0.8209	0.8197	0.782
Extra Trees Classifier	0.8112	0.8112	0.8125	0.8093	0.7699
Quadratic Discriminant Analysis	0.8076	0.8076	0.8153	0.8046	0.7645
Gradient Boosting Classifier	0.7694	0.7694	0.774	0.7708	0.7213
Logistic Regression	0.7384	0.7384	0.7553	0.7428	0.6869
Linear Discriminant Analysis	0.7157	0.7157	0.744	0.7228	0.6614
Ridge Classifier	0.7025	0.7025	0.726	0.7059	0.6457

SVM - Linear Kernel	0.6846	0.6846	0.7118	0.6893	0.6232
Naive Bayes	0.583	0.583	0.6301	0.5902	0.5064
Decision Tree Classifier	0.5448	0.5448	0.5565	0.5484	0.4534
Ada Boost Classifier	0.2342	0.2342	0.3691	0.2108	0.1419
Dummy Classifier	0.1983	0.1983	0.0393	0.0656	0
